# Spatiotemporal Analysis of the Carbon Footprint of Soybean Production in China Based on Life Cycle Assessment

**DOI:** 10.3390/foods15111979

**Published:** 2026-06-02

**Authors:** Guoguo Ning, Fanhao Yang, Jianya Zhao, Shu Wang

**Affiliations:** 1 Joint Institute of Jinan University and University of Birmingham, Jinan University, 855 Xingye E. Ave., Guangzhou 511436, China; nggyhh@stu2023.jnu.edu.cn (G.N.); dukeyoung@stu2024.jnu.edu.cn (F.Y.); 2 School of Mathematics, University of Birmingham, Edgbaston, Birmingham B15 2TT, UK; 3 School of Economics, Jinan University, 601 W. Huangpu Ave., Guangzhou 510632, China

**Keywords:** soybean production, carbon footprint, life cycle assessment, agricultural carbon emissions, low-carbon agriculture

## Abstract

Against the backdrop of global climate change and the “dual carbon” goals, the issue of agricultural greenhouse gas emissions has garnered increasing attention. As a major grain and oilseed crop in China, carbon emissions from soybean production have a significant impact on the green and low-carbon development of agriculture. Although research on agricultural carbon footprints has grown in recent years, existing studies have largely focused on single regions or specific stages of crop production, and analyses of the carbon footprint of production systems in China’s major soybean-producing regions remain relatively limited. This study employs the Life Cycle Assessment (LCA) methodology to calculate and analyze the carbon footprint of soybean production systems across China’s 10 major soybean-producing provinces, utilizing agricultural input data from 2014 to 2023. The study establishes a carbon footprint accounting system based on two key aspects: carbon emissions from agricultural inputs and soil N_2_O emissions. It further analyzes the temporal trends, regional variations, and contribution characteristics of each component within the carbon footprint. The results indicate that the average carbon footprint of soybean production in China is approximately 528 kg CO_2_eq/ha (ranging from 273 to 855) and 0.25 CO_2_eq/kg of soybean (ranging from 0.13 to 0.46). Specifically, the carbon footprint per unit of area and yield declined simultaneously, indicating a continuous improvement in the low-carbon efficiency of soybean production. Spatially, there are significant regional differences in the carbon footprint of soybean production. Henan, Anhui, and Inner Mongolia have relatively low carbon footprints, while Shaanxi and Shanxi have relatively high levels. In terms of composition, chemical fertilizer inputs and soil N_2_O emissions are the primary sources of the carbon footprint in soybean production, with chemical fertilizer inputs being the largest source, accounting for approximately 40–60%, and soil N_2_O emissions being the second major source. Overall, differences among regions in natural conditions, agricultural input structures, and production methods result in distinct regional characteristics in the carbon footprint composition. The findings of this study provide a scientific basis for the low-carbon transition of China’s soybean production system and serve as a reference for the formulation of policies related to green agricultural development.

## 1. Introduction

### 1.1. Background on Agricultural Emissions Reductions and Carbon Emissions from Food Systems

The continuous accumulation of greenhouse gas emissions driven by human activities has made climate change one of the core challenges facing global sustainable development. Existing research generally agrees that the food system has become a major source of global greenhouse gas emissions, accounting for approximately 21–37% of total global emissions; this range encompasses multiple stages, including agricultural production, land-use change, storage and transportation, processing, retail, and consumption [1,2]. Among these, agricultural production itself remains one of the primary sources of emissions [3]. As global climate governance continues to strengthen, countries worldwide are advancing emission reduction policies and industrial transformation in line with the goals of the Paris Agreement. Consequently, agricultural emission reduction has evolved from a purely environmental issue into a comprehensive challenge that encompasses food security, resource efficiency, and rural sustainable development [4].

As a major global agricultural producer and a significant emitter of greenhouse gases, China has clearly set forth its “dual carbon” goals of “reaching carbon peak by 2030 and achieving carbon neutrality by 2060” [5]. In line with this strategic deployment, the green and low-carbon transition in the agriculture and rural sectors has been incorporated into the national emissions reduction framework. In 2022, the Ministry of Agriculture and Rural Affairs and the National Development and Reform Commission jointly issued the “Implementation Plan for Emissions Reduction and Carbon Sequestration in Agriculture and Rural Areas,” explicitly calling for the coordinated advancement of stable agricultural production and supply [6], emissions reduction and carbon sequestration, resource conservation, and ecological protection to achieve the synergistic development of food security and green transformation [7]. Consequently, under the premise of ensuring national food security, systematically identifying the carbon emission characteristics, key emission stages, and emission reduction potential in the production processes of major crops has become a critical research direction for implementing the “Dual Carbon” strategy.

### 1.2. The Strategic Role of Soybean in China’s Food Security and Low-Carbon Agricultural Transition

As a cornerstone of China’s grain and oilseed security, soybeans occupy a uniquely strategic position by underpinning the nation’s edible oil processing and livestock feed industries. Currently, the crop is the focal point of the “Soybean Revitalization Plan,” a national initiative designed to bridge the significant supply–demand gap and reduce external dependency by aggressively expanding cultivated areas and enhancing productivity. While this strategic push is essential for stabilizing the domestic protein industry chain, such a large-scale intensification necessitates a critical evaluation of the associated environmental trade-offs [3,8].

Under the “Soybean Revitalization Plan,” China has significantly enhanced its domestic supply capacity, with production reaching a record 20.84 million metric tons in 2023 [9]. This achievement was driven by expanding the national planting area to 10.47 million hectares and increasing average yields to 1990.5 kg/hathrough policy incentives and variety improvements. However, this growth occurs amidst a strategic shift toward a green agricultural transition, requiring soybean production to synergize food security with Carbon footprint mitigation. Consequently, identifying regional variations in Carbon footprints and characterizing input structures is critical for formulating evidence-based pathways that align production targets with environmental sustainability.

### 1.3. Literature Review

The carbon footprint has become an important tool for assessing greenhouse gas emissions from agricultural production systems. Derived from the concept of the ecological footprint, it is typically defined as the total greenhouse gas emissions generated by a product or activity throughout its life cycle, expressed in carbon dioxide equivalents (CO_2_e) [10,11]. In the agricultural sector, carbon footprint analysis is typically based on the Life Cycle Assessment (LCA) methodology. By establishing a system boundary that spans “from input production to field harvest,” it systematically accounts for the production and use of inputs such as chemical fertilizers, pesticides, and diesel, as well as greenhouse gas emissions from farmland soils [12,13]. With the continuous advancement of research on green agricultural development and climate change, the LCA method has been widely applied to the assessment of greenhouse gas emissions from crop production systems and has gradually become an important analytical framework for low-carbon agriculture research.

In research on the carbon footprint of crops, the existing literature has primarily focused on staple crops such as rice, wheat, and corn, while studies on soybean production systems are relatively scarce. Existing research indicates that the carbon emissions from soybean production systems are generally lower than those of some high-input staple crops; however, emission levels are still significantly influenced by regional agricultural production conditions. Based on Chinese agricultural statistics, Cheng et al. (2015) [14] found that the carbon footprint per unit of soybean production varies significantly across different regions, indicating that regional agricultural input structures and production conditions have a significant impact on carbon emissions. Consequently, the regional characteristics of carbon emissions from soybean production and their driving mechanisms have gradually become important topics in agricultural carbon footprint research.

With regard to specific emission sources, numerous studies have shown that the production of agricultural inputs and N_2_O emissions from farmland soils are the primary sources of greenhouse gas emissions in soybean production. Maciel et al. (2016) [15] conducted a life cycle assessment of soybean production systems in southern Brazil and found that, excluding land-use change, the carbon footprint of soybean production was approximately 0.35 kg CO_2_e kg^−1^, with lime application, fertilizer production, and farm machinery operations being the primary emission sources. Similarly, a study by Arrieta et al. (2018) [16] also found in a study of soybean production systems across different agricultural regions in Argentina that the production of agricultural inputs and soil N_2_O emissions constitute the bulk of the carbon footprint, and that emission levels vary significantly among different agricultural regions.

In recent years, several studies have further examined the mechanisms of greenhouse gas emissions from soybean production systems from the perspective of agricultural ecological processes. A study by Della Chiesa et al. (2024) found that in a corn–soybean rotation system [17], the soybean phase may still generate high N_2_O emissions, contributing up to approximately 40% of the total emissions across the entire rotation system. This indicates that leguminous crops are not entirely “low-emission” crops, and their carbon footprint remains significantly influenced by soil nitrogen cycling processes. Furthermore, climatic conditions and yield fluctuations can also significantly impact the soybean carbon footprint. Giusti et al. (2023) found that during extreme drought years, soybean carbon footprints can increase by more than 200% due to a significant rise in emissions per unit of production resulting from yield declines, indicating that climate shocks may substantially alter the carbon emission intensity of crop production systems [18].

In addition to natural conditions, agricultural management practices are also a key factor influencing the carbon footprint of soybeans. Castanheira and Freire (2013) noted that differences in tillage systems can significantly alter emission levels in soybean production systems [19]; no-till or conservation tillage typically reduces fuel consumption by machinery and increases soil carbon stocks, thereby lowering the overall carbon footprint. Related studies have also found that, under certain conditions, the introduction of cover crops and conservation tillage practices can significantly reduce lifecycle greenhouse gas emissions from soybean production systems by decreasing energy inputs and increasing soil carbon sinks [20].

As research on agricultural sustainability continues to advance, international studies on crop life cycle assessment (LCA) and carbon footprints have gradually expanded from calculations focused on single crops or specific regions to comprehensive evaluation frameworks that integrate multi-crop, multi-scale, and uncertainty analyses. Existing research indicates that the carbon footprint of crops is not only directly influenced by the consumption of agricultural inputs and greenhouse gas emissions from field operations but is also closely related to methodological considerations such as system boundary definition, the selection of functional units, and whether changes in soil carbon are included [21]. At the national level, Yan et al. (2015) conducted a systematic calculation of the carbon footprint of China’s grain crop production systems based on farmer survey data, identifying chemical fertilizer production and application, energy consumption by agricultural machinery, and N_2_O emissions from farmland soils as the primary sources of greenhouse gas emissions in crop production systems [22]. Furthermore, Chen et al. (2021) adopted a “cradle-to-farm-gate” life cycle analysis framework to conduct a unified assessment of the carbon footprints of 16 major crop production systems in China, finding that fertilization and irrigation are the key processes influencing differences in carbon footprints across crops [23]. Song et al. (2023) estimated the carbon footprints of major food crops and their associated uncertainties at the prefecture-level city scale, emphasizing that spatial heterogeneity and parameter uncertainties significantly influence regional comparison results [24]. Regarding soybean production systems, research by Castanheira and Freire (2013) further demonstrated that different tillage regimes and land-use change scenarios can significantly alter the greenhouse gas emission levels and carbon footprint intensity of soybean production systems [19].

In summary, existing research has systematically elucidated the mechanisms underlying crop production carbon footprints from multiple perspectives, including agricultural input structures, field ecological processes, regional variations, and the optimization of management practices. Focusing on soybean production systems, existing research generally agrees that chemical fertilizer inputs, N_2_O emissions from farmland soils, and energy consumption by agricultural machinery are the primary sources of carbon emissions. At the same time, studies also indicate that differences in tillage practices, as well as the heterogeneity of regional resource endowments and production conditions, are key factors leading to spatial variations in the carbon footprint of soybean production across different regions.

### 1.4. Research Gaps and the Objectives of This Study

Although previous studies have laid a solid foundation, three critical research gaps remain in the assessment of soybean carbon footprints. First, in terms of research scale, existing studies on the carbon footprint of soybean production have largely focused on individual farms, experimental plots, or local regions, such as the Northeast region or typical production areas in Heilongjiang Province [15,25]. These studies provide important insights into the carbon emission patterns of on-farm production processes, and their findings are often limited by regional conditions and differences in production practices, making it difficult to reflect the overall variations among China’s major soybean-producing regions.

Second, in terms of research scope, few existing studies have conducted systematic comparative analyses of multiple major production regions within a unified accounting framework. Soybean production in China is characterized by a distinct regional concentration, primarily distributed across the Northeast Plain, the Huang-Huai-Hai Plain, and certain peripheral production areas in the north. Due to significant differences among regions in terms of climatic conditions, soil types, agricultural input structures, and levels of agricultural mechanization, the carbon emission intensity of soybean production systems may exhibit marked spatial heterogeneity [14,16]. Therefore, conducting a systematic comparison of the carbon footprints of soybean production across different regions at the regional scale is of great significance for identifying major emission sources within agricultural production systems and assessing emission reduction potential. Differences among studies regarding system boundary definitions, emission factor selection, and sources of agricultural input data have, to some extent, affected the comparability of results across studies [13]. Therefore, conducting cross-regional comparative studies within a unified life-cycle accounting framework is crucial for revealing carbon emission differences among various production systems.

Third, in terms of the time scale, most studies typically estimate carbon footprints based on data from a single year or short-term data; research on trends in soybean production carbon footprints over longer time series remains relatively limited. In particular, at the level of China’s major soybean-producing regions, there is a lack of studies that systematically analyze the spatiotemporal characteristics of carbon footprint changes based on long-term statistical data.

To fill these gaps, this study employs a unified life-cycle framework to analyze soybean production across China’s 10 major provinces from 2014 to 2023. The primary objectives of this study include: (1) quantify spatiotemporal trends, revealing how soybean Carbon Footprint has evolved over a decade of environmental and policy shifts; (2) characterize regional heterogeneity, identifying carbon intensity differences between primary and marginal production zones; and (3) elucidate emission structures, determining the dominant emission drivers to provide a scientific basis for differentiated, low-carbon agricultural policies.

Through a systematic comparison and long-term trend analysis of carbon footprints across China’s major soybean-producing regions, this study aims to reveal the differences in carbon emission structures among these regions and the patterns of their changes, thereby providing a scientific basis for promoting the low-carbon transition of China’s soybean production system and offering guidance for the formulation of policies related to green agricultural development [3].

## 2. Materials and Methods

### 2.1. Study Area

**Figure 1:**
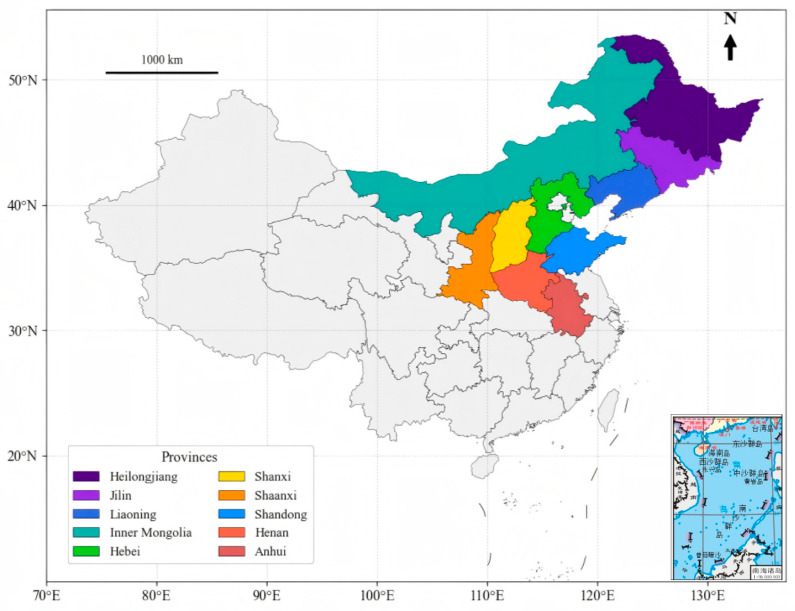
Geographical Location of Major Soybean-Producing Provinces in China.

The major soybean-producing regions in China primarily include Hebei, Shanxi, Inner Mongolia, Liaoning, Jilin, Heilongjiang, Anhui, Shandong, Henan, and Shaanxi, with the geographical locations of these provinces shown in Figure 1. The study area covers the major soybean-producing regions of Northeast China, the Huang-Huai-Hai Plain, and the northwestern marginal production areas, spanning distinct climatic zones and resource endowment conditions. Therefore, it provides an appropriate basis for comparing differences in soybean carbon footprints across different production systems. These provinces share common geographical and climatic characteristics, with most areas located in temperate and subarctic climates. They experience distinct seasons, with cold and dry winters and warm, humid summers, conditions that are favorable for soybean growth. In addition, the predominant soil types in these regions are black soil and gray-brown soil, both of which are fertile with high organic matter content. The water resources are relatively abundant, providing favorable natural conditions for soybean cultivation.

**Figure 2:**
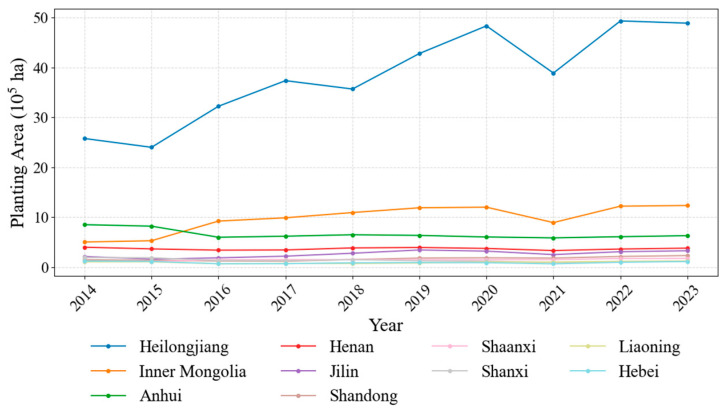
Interannual Variation in Soybean Planting Area in China’s Major Producing Regions from 2014 to 2023.

Figure 2 illustrates the annual variation in soybean planting areas across the major soybean-producing provinces in China from 2014 to 2023 [26]. Heilongjiang Province, as the core soybean production area in China, dominates the planting area, significantly surpassing other provinces. The soybean planting area in Heilongjiang has shown a generally fluctuating upward trend, increasing from approximately 2,500,000 ha in 2014 to nearly 5,000,000 ha in 2023, almost doubling. Despite brief declines in 2015 and 2021, the overall expansion momentum remains strong. Inner Mongolia ranks second in terms of planting area, with a clear growth trend. Although its total area lags behind Heilongjiang, the growth rate is relatively high, indicating substantial potential for expansion. In contrast, provinces such as Henan, Hebei, and Shandong have relatively smaller soybean planting areas, mostly remaining below 500,000 ha in most years. The annual variation in these provinces is minimal, and the growth curves tend to be flatter, suggesting that the planting scale in Huang-Huai-Hai and other regions remains relatively stable, with no significant expansion or contraction.

### 2.2. Data Source and Processing

In this study, the agricultural input data for the soybean production in 10 provinces is sourced from the “National Agricultural Product Cost and Benefit Data Compilation” from 2014 to 2023 [27]. The price data for pesticides and diesel is sourced from the “Market Price Changes of Important Production Materials in the Circulation Field” published by the National Bureau of Statistics from 2014 to 2023 (https://www.stats.gov.cn). The data of China’s Major Soybean Producing Regions is also sourced from the annual data of the National Bureau of Statistics from 2014 to 2023 (https://www.stats.gov.cn). Specifically, a conversion-based approach was adopted in this study to indirectly estimate mechanical diesel input. The mechanical diesel cost was calculated as mechanical operation cost × 0.3 [28]. This coefficient represents an empirical proportion of fuel cost in mechanical operation expenditure. Owing to the lack of direct data on diesel consumption and pesticide use, mechanical diesel consumption was further estimated as mechanical diesel cost divided by diesel price, while pesticide use was estimated as pesticide cost divided by pesticide price. In addition, due to data availability constraints, nationally averaged annual prices were uniformly applied for the conversion. Although this treatment helps ensure comparability across different years and regions, it may still introduce some estimation bias because it does not fully reflect interprovincial differences in prices, regional mechanization levels, or operation modes.

### 2.3. Methods

**Figure 3:**
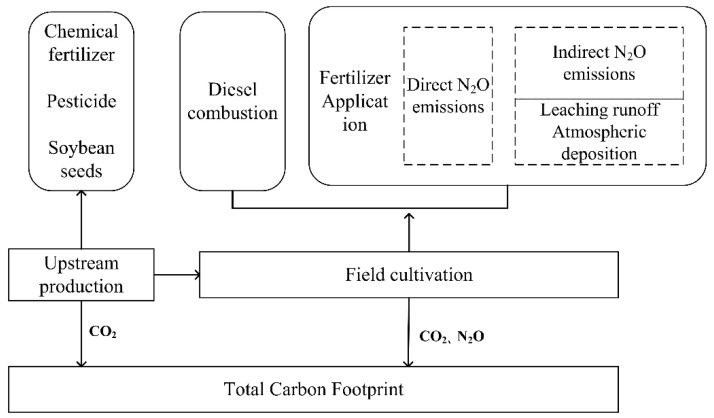
System Boundaries of the Life Cycle Assessment Method for Soybean Carbon Footprint.

This study uses the Life Cycle method to calculate the carbon footprint of the soybean production system. The system boundary for soybean production is defined as the lifecycle stage from sowing to harvest, and the carbon footprint accounting boundary includes greenhouse gas emissions from agricultural inputs and soil N_2_O emissions. Soil N_2_O emissions consist of direct emissions resulting from nitrogen fertilizer application, as well as indirect emissions from atmospheric deposition, runoff, and leaching. Greenhouse gas emissions from agricultural inputs mainly result from the use of seeds, pesticides, fertilizers, and diesel (Figure 3).

(1) Carbon emissions from agricultural inputs: The agricultural inputs during the life cycle of soybean primarily include eight inputs: seeds, chemical fertilizers (nitrogen, phosphorus, potassium, compound fertilizers), diesel, and pesticides. These inputs are converted into carbon emission equivalent (CE). The carbon footprint calculation formula is as follows:

(1) $$CE_{input}=\sum_{i=1}^{n}\left(Input_{i}\times EC_{i}\right)$$

where ${CE}_{input}$ is the carbon emission from agricultural inputs (kg/ha); $Input_{i}$ refers to the input amount of the i-th agricultural input product $\left(kg\right)$; and n refers to the number of agricultural input types. $EC_{i}$ refers to the emission coefficient of the i-th agricultural input product, see Table 1.

(2) Soil N_2_O emissions: These mainly include the direct N_2_O emissions caused by nitrogen fertilizer application, as well as the indirect N_2_O emissions caused by atmospheric deposition and leaching runoff. The calculation formulas are as follows:

(2) $$NF_{1}=N_{input}\times NE_{1}\times \frac{44}{28}$$

(3) $$NF_{2}=N_{input}\times NE_{2}\times NE_{a}\times \frac{44}{28}$$

(4) $$NF_{3}=N_{input}\times NE_{3}\times NE_{b}\times \frac{44}{28}$$

(5) $$CE_{N_{2}O}=(NF_{1}+NF_{2}+NF_{3})\times 298$$

In Formula (2), ${NF}_{1}$ represents the N_2_O emissions from the application of chemical fertilizers (kg/ha); $N_{input}$ represents the amount of fertilizer applied in agriculture (kg/ha); $NE_{1}$ is the direct emission factor of N_2_O from the application of chemical fertilizers, assigned a value of 0.01 according to the 2006 IPCC guidelines [34]; and 44/28 is the ratio of molecular weight between N_2_O -N and N_2_O. In Formulas (3) and (4), ${NF}_{2}$ represents the N_2_O emissions from atmospheric deposition (kg/ha); ${NF}_{3}$ represents the N_2_O emissions from leaching (kg/ha); $NE_{a}$ represents the emission factor for agricultural soil and NO_X_ emission rate; $NE_{b}$ represents the emission factor for leaching and runoff rate. According to the recommendation in the “National Climate Change Guidelines for the Greenhouse Gas Emission Inventory” by the National Climate Change Office [35], $NE_{a}$ is taken as 10%, and $NE_{b}$ is taken as 20%. $NE_{2}$ is the direct emission factor of N_2_O from atmospheric deposition (0.01) and $NE_{3}$ represents the direct emission factor of N_2_O from leaching (0.01). Formula (5) converts the emitted amount of N_2_O into CO_2_ equivalent. $CE_{N_{2}O}$ represents the N_2_O emissions from direct and indirect emissions of CO_2_, in kg/hm^2^ (calculated as CO_2_ equivalent); 298 is the global warming potential for a 100-year time horizon, based on the IPCC Fourth Assessment Report (AR4) [36,37,38,39,40].

(3) Total Carbon Footprint:

(6) $$CF_{area}=CE_{input}+CE_{N_{2}O}$$

(7) $$CF_{yield}=CF_{area}/Y$$

where ${CE}_{input}$ is the carbon emission from agricultural inputs (kg CO_2_-eq/ha); $CF_{area}$ is the total carbon footprint per unit area for soybean production (kg CO_2_-eq/ha); Y is the soybean yield per unit area (kg/ha); and $CF_{yield}$ is the carbon footprint per unit yield of soybean production (kg CO_2_-eq/kg).

Data processing and statistical analyses were conducted using R version 4.5.3. To evaluate the differences between groups, Student’s *t*-test was employed, with statistical significance defined as *p* < 0.05. Box plots were used to visualize the distribution of area-scaled and yield-scaled carbon footprints across different provinces and years, showing the median, 25th/75th percentiles, and whiskers. Regression analysis was applied to fit the relationship between Years and Carbon Footprint per unit area and Carbon Footprint per unit yield, and the accuracy of the fits was assessed using the R^2^ value.

## 3. Results

### 3.1. Trends in Carbon Footprint over Time in China’s Major Soybean-Producing Regions

**Figure 4:**
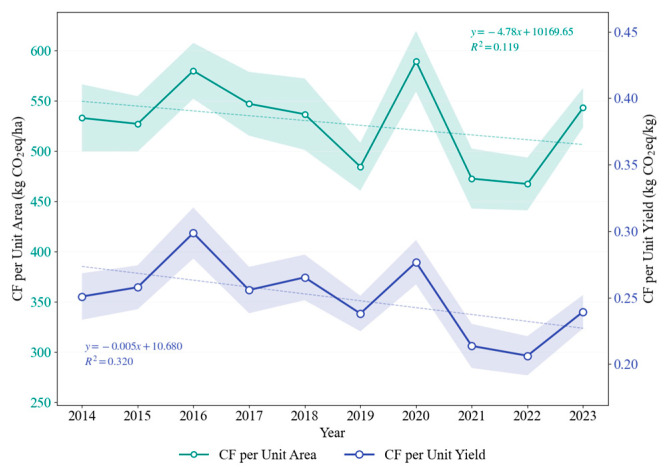
Annual carbon footprint intensity of agricultural production: per area and per unit yield from 2014 to 2023. Note: The shaded boundaries around the lines represent Mean ± 0.5 × SEM, where SEM refers to the standard error of the mean, and reflect the spatial heterogeneity among the ten major soybean-producing provinces.

According to the analysis, both indicators exhibited a fluctuating downward trend over the study period, showing a synchronized pattern of “rise–decline–rise–decline.” Specifically, a linear regression trend test (Ordinary Least Squares) was employed to evaluate the temporal changes in Carbon Footprint. The results indicate that the average carbon footprint of soybean production in China is approximately 528 kg CO_2_eq/ha (ranging from 273 to 855) and 0.25 CO_2_eq/kg of soybean (ranging from 0.13 to 0.46). As illustrated in Figure 4, the Carbon Footprint per unit area exhibited a numerical decline at an average rate of 4.78 kg CO_2_eq/ha per year (y=−4.78x+10,169.65). However, this change did not reach statistical significance (p=0.33). The value peaked in 2020 (589.16 kg CO_2_eq/ha) and reached its lowest point in 2022 (467.14 kg CO_2_eq/ha). In contrast, the Carbon Footprint per unit yield showed a marginally significant downward trend with a slope of −0.005 (p=0.09). The highest value of Carbon Footprint per unit yield was observed in 2016 (0.30 kg CO_2_eq/kg), followed by a sharp decline of 22.8% between 2020 and 2021, driving it to its lowest point in 2022.

### 3.2. Spatiotemporal Heterogeneity in Carbon Footprints

To provide a holistic assessment of soybean sustainability, it is essential to distinguish between the roles of the two functional units used in this study. Carbon Footprint per unit area is the preferred metric for evaluating the direct environmental pressure on local ecosystems and is highly relevant for regional land management policies [41]. However, it may overlook the efficiency of resource utilization. Conversely, the Carbon footprint per unit yield effectively quantifies low-Carbon production efficiency, serving as a more rigorous indicator for balancing environmental mitigation with food security objectives [42]. Its limitation lies in its sensitivity to annual yield fluctuations caused by non-management factors such as extreme weather [43]. By employing both units, this study provides a more holistic assessment of the sustainability of soybean production.

**Figure 5:**
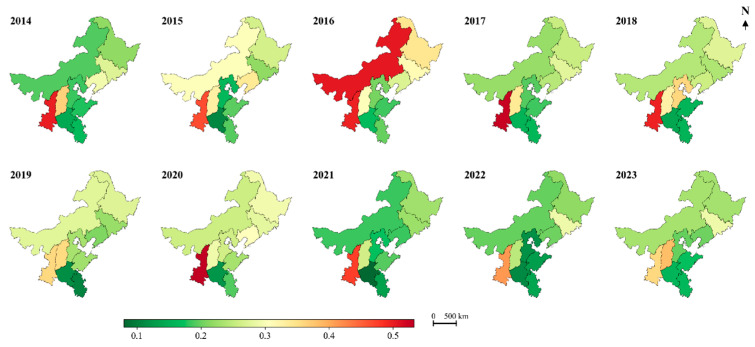
Spatial Distribution of Carbon Footprint per Unit Yield in China’s Major Soybean Producing Regions from 2014 to 2023 (kg CO_2_ eq/kg).

Figure 5 visually illustrates the spatiotemporal evolution of carbon footprints across China’s primary soybean-producing regions from 2014 to 2023. The time-series maps indicate that Carbon intensity underwent a non-linear trajectory, shifting from an initial period of relative stability to a phase of localized increases, before entering a stage of broad-based reduction and structural optimization. A notable spatial synchronization emerged in 2016, with both the Northeast and Huang-Huai-Hai regions exhibiting peak Carbon footprint values, as indicated by the prevalence of high-intensity markers on the map. By 2020, the distribution pattern transitioned toward a distinct north–south divergence; while emission intensity in the Northeast began to become moderate, localized rebounds were observed in provinces such as Henan, Anhui, and Shaanxi. In the final period (2021–2023), a consistent shift toward lower Carbon footprint values occurred nationwide, signaling a widespread improvement in Carbon efficiency. Our calculated Carbon footprint estimates (467.14–589.16 kg CO_2_eq/ha) align with existing literature for intensive soybean systems in Northeast China, though they predictably exceed the values reported for low-input organic systems.

**Figure 6:**
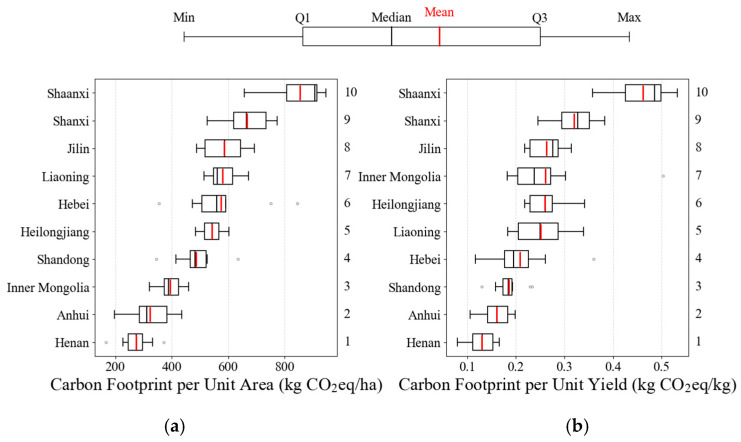
Carbon Footprint in Different Provinces of China’s Major Soybean-Producing Regions from 2014 to 2023 (kg CO_2_ eq/kg). (**a**) Carbon Footprint per unit area by province, (**b**) Carbon Footprint per unit yield by province.

From a geographical perspective, the carbon footprint across China’s major soybean-producing regions exhibits significant zonal patterns and spatial heterogeneity. According to the provincial analysis in Figure 6, Henan, Anhui, and Inner Mongolia maintain the lowest mean Carbon Footprint per unit area, with Henan averaging approximately 273 kg CO_2_eq/ha. In sharp contrast, Shaanxi and Shanxi exhibit the highest intensities, exceeding 750 kg CO_2_eq/ha—roughly 2.5 times that of Henan. When shifting the metric to Carbon Footprint per unit yield (Figure 6, right panel), provincial rankings undergo a notable realignment. While Henan and Anhui retain their low-emission advantage, Inner Mongolia’s ranking for carbon efficiency declines despite its low area-based inputs. Conversely, Shandong’s relative position improves in terms of yield-based efficiency. Furthermore, the boxplot analysis reveals that Henan, Heilongjiang, and Anhui possess relatively short interquartile ranges (IQRs), indicating stable interannual production performance. In contrast, Shaanxi and Hebei display longer boxes and prominent outliers, signaling high interannual variability in carbon trajectories.

### 3.3. Contributions of Each Component to the Carbon Footprint

**Figure 7:**
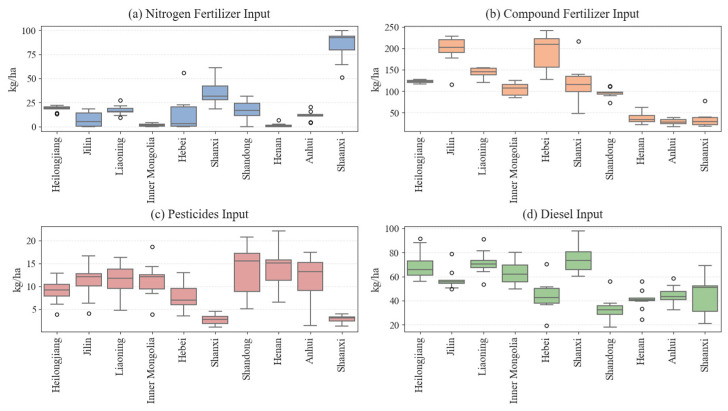
Spatio-temporal variations in major agricultural physical inputs for soybean production across China’s key producing provinces (2014–2023). Note: The boxplots illustrate inter-provincial differences and inter-annual fluctuations in: (**a**) Nitrogen fertilizer input (kg/ha); (**b**) Compound fertilizer input (kg/ha); (**c**) Pesticide input (kg/ha); and (**d**) Agricultural diesel input (kg/ha). The horizontal line within each box represents the median, while the whiskers and outliers reflect the temporal variability within each province over the study period.

The spatiotemporal variations in agricultural physical inputs across China’s soybean-producing regions reveal a highly heterogeneous landscape of farming intensities from 2014 to 2023. As illustrated in Figure 7, nitrogen and compound fertilizers remain the most dominant yet variable inputs, with Shaanxi emerging as a significant outlier in pure nitrogen application—averaging nearly 90 kg/ha (Figure 7a)—while Jilin and Hebei rely heavily on compound fertilizers at rates exceeding 200 kg/ha (Figure 7b). Beyond fertilization, pesticide usage (Figure 7c) shows the highest inter-annual volatility in Shandong, Henan, and Anhui, as evidenced by the extended whiskers and large interquartile ranges in the boxplots. In terms of mechanical energy, agricultural diesel consumption (Figure 7d) maintains a consistently high and stable level in the mechanized frontiers of Heilongjiang, Liaoning, Inner Mongolia, and Shanxi, with medians consistently exceeding 60 kg/ha.

**Figure 8:**
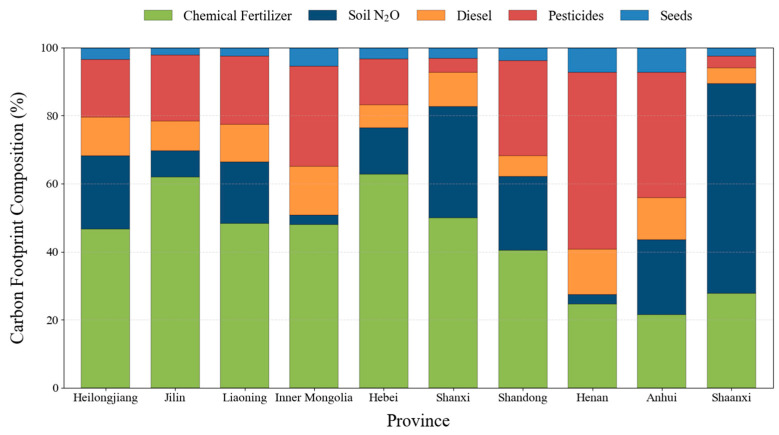
Contribution of Different Components to the Carbon Footprint of Soybean Production in China’s Major Soybean-Producing Provinces from 2014 to 2023.

These agricultural input patterns directly shape the composition ratios of the carbon footprint across provinces, as illustrated in Figure 8. Overall, although chemical fertilizers and soil-derived N_2_O emissions remain the core contributors to the carbon footprint nationwide, their structural characteristics exhibit significant spatial heterogeneity, which can be primarily categorized into the following typical patterns:

(1) The “Fertilizer-driven” pattern in Northeast and North China: Directly driven by the massive compound fertilizer inputs depicted in Figure 7b, the Northeastern region (Heilongjiang, Jilin, Liaoning) and the North China region (Hebei, Shanxi) exhibit the highest contribution rates of chemical fertilizers to the carbon footprint, ranging from 40% to 60%.
(2) The “N_2_O-dominant” pattern in Shaanxi: Shaanxi exhibits a unique emission profile where soil-derived N_2_O emissions account for a staggering 61.7% of the total Carbon Footprint, the highest among all studied provinces. This disproportionate share directly corresponds to the high nitrogen input levels observed in Figure 7a.
(3) The “Pesticide-intensive” pattern in Henan and Anhui: The Carbon Footprint composition in these two provinces is uniquely weighted toward pesticides, with contribution rates reaching 51.9% and 36.9%, respectively. This is consistent with the high median pesticide inputs and wide inter-annual fluctuations observed in the raw data.
(4) The “Diesel-prominent” pattern in Inner Mongolia: The proportion of diesel emissions in Inner Mongolia is noticeably higher than in other provinces. It is noteworthy that, when cross-referencing the absolute quantity data in Figure 7d, Inner Mongolia’s physical diesel input is actually comparable to that of Heilongjiang. However, owing to its relatively low levels of fertilizer and pesticide inputs, the “relative proportion” of diesel in the total carbon footprint is significantly amplified.

Across all provinces, the carbon contribution from the seed production stage remained marginal and stable, consistently accounting for less than 8% of the total emissions, with negligible inter-provincial disparities.

## 4. Discussion

### 4.1. Analysis of Temporal Fluctuations and Trend Validation

The carbon footprint of China’s major soybean-producing regions from 2014 to 2023 exhibited a “fluctuating decline” pattern, signaling a strategic transition toward low-carbon intensification. The anomalous peaks observed between 2019 and 2022 were likely driven by the socioeconomic disruptions of the COVID-19 pandemic and the increased frequency of extreme weather events in major soybean-producing regions. Evidence suggests that labor shortages during the pandemic acted as a catalyst for a “labor-input substitution” effect [44,45,46], where farmers increased chemical pesticide applications to compensate for reduced manual field management capacity. Simultaneously, frequent extreme precipitation events likely triggered nutrient leaching, necessitating supplemental fertilization to offset losses, which artificially inflated the Carbon Footprint during this window. This observation aligns with existing theories that global crises often force temporary shifts in agricultural input structures to maintain stability.

When validating the reliability of our calculated values (ranging from 467.14 to 589.16 kg CO_2_eq/ha), our estimates prove consistent with the ranges reported for intensive soybean systems in Northeast China. However, our values are notably higher than those found in low-input organic systems, highlighting the significant role of regional management intensity and climatic stability in shaping carbon trajectories. To further ensure accuracy, we systematically compared our estimates with previous peer-reviewed literature in Tables S1 and S2 (see in Supplementary Materials). At a macro-level, our provincial estimates for regions such as Heilongjiang strongly align with the findings of Chen et al. (2024) and Xiu et al. (2025), which were conducted at a similar provincial scale and temporal scope [25,47]. In contrast, cross-scale comparisons reveal evident “limitations in comparability.” For instance, the discrepancies with Chu et al. (2021) are primarily attributed to their focus on a micro-regional scale (Nenjiang Farm) [48], where unique soil-climatic conditions and highly intensive management make its results unrepresentative of the regional average.

Furthermore, the compositional characteristics of the Carbon Footprint (Table S2) emphasize the importance of temporal scope in comparative analysis. For Northeast China, our study largely overlaps with Chen et al. (2024) and Xiu et al. (2025), all concluding that chemical fertilizer inputs and soil background N_2_O emissions are the primary contributors [25,47]. However, regarding Henan Province, a significant discrepancy exists between our results and those of Li et al. (2025), driven by temporal asymmetry [49]. Li et al. (2025) evaluated a prolonged time series (2004–2022) capturing earlier periods of traditional, extensive agriculture that relied on excessive chemical inputs [49]. Consequently, their estimated average contribution rate of chemical fertilizers (63.76%) is substantially higher than our study’s estimate (24.59%) for the most recent decade (2014–2023). This remarkable cross-temporal contrast not only rationally explains the numerical variations but also serves as compelling collateral evidence of the substantial achievements of China’s recent “Chemical Fertilizer Reduction and Efficiency Enhancement Action” in successfully decoupling soybean production from high carbon intensity [50,51].

### 4.2. Regional Heterogeneity and the “Yield Dilution Effect”

The spatial disparity in Carbon Footprint is not merely a reflection of absolute input volumes but is deeply rooted in the complex interplay between topographical constraints and resource use efficiency [52,53]. Our results reveal that provinces with consistently high Carbon Footprint per unit area, such as Shaanxi and Shanxi, suffer from a significant “efficiency penalty” imposed by non-plain terrains. In these loess plateau and hilly regions, rugged terrain diminishes the fuel efficiency of agricultural machinery and increases the energy intensity of field operations, preventing the use of high-horsepower, energy-efficient tractors.

Conversely, the performance of high-input but high-efficiency provinces, exemplified by Shandong, underscores the critical role of the “Yield Dilution Effect.” While these regions maintain high levels of fertilizer and energy inputs to support intensive cultivation, their superior yields—often exceeding the national average by a significant margin—effectively “dilute” the carbon cost associated with each kilogram of soybean produced. This observed mechanism aligns with the “Sustainable Intensification” paradigm currently debated in high-level agricultural literature [54]. We conclude that high-yielding cultivation acts as a powerful carbon-mitigation mechanism through the maximization of resource use efficiency. In these high-productivity systems, the enhanced carbon-sequestration capacity of the crop, paired with optimized nutrient uptake, creates a physiological buffer that dampens the carbon intensity of production.

Crucially, the divergent Carbon trajectories between the hilly interior and the plain-based intensive systems highlight a “topographical-efficiency trap.” In regions where geographical constraints prevent the optimization of input–output ratios, the Carbon footprint remains stubbornly high regardless of moderate input reductions. This suggests that for China’s soybean sector, the inherent Carbon trajectory is governed by a decoupling mechanism where yield gains must outpace input growth to achieve a net reduction in intensity. The Carbon intensity per unit yield of soybean is, therefore, less a function of total input application and more a reflection of the landscape’s capacity to facilitate the conversion of these inputs into biomass.

### 4.3. Uncertainty and Limitations

While this study systematically characterizes the carbon footprint of China’s soybean production, several inherent limitations must be acknowledged to contextualize the findings. In terms of data acquisition, the input quantities for pesticides and diesel were primarily derived via cost-backward estimation due to the constraints of large-scale statistical accessibility. Although this method is widely accepted in macro-level agricultural studies, the temporal and spatial fluctuations in market prices, coupled with regional variations in agricultural subsidies, may introduce subtle discrepancies between estimated values and actual physical consumption. Consequently, this methodological reliance represents a potential source of uncertainty regarding the absolute precision of the Carbon Footprint calculations.

Furthermore, the reliance on nationally standardized emission factors—necessitated by the current lack of high-resolution, localized life cycle inventory databases—may partially mask the influence of specific regional conditions. In reality, the heterogeneous interplay of soil characteristics, climatic parameters, and agricultural machinery efficiency across China’s diverse provinces likely results in distinct emission profiles that uniform parameters cannot fully capture. This suggests that while the observed trends remain robust, the localized intensity might be slightly over- or under-estimated in specific high-latitude or hilly terrains.

Regarding the definition of the system boundary, this study prioritized core cultivation processes to ensure data consistency, which led to the exclusion of certain secondary stages. Specifically, electricity consumption for irrigation and post-harvest straw management—such as N_2_O emissions from straw incorporation or open-air burning—were not incorporated into the current boundary. Moving forward, future research should prioritize extensive field-level surveys to develop localized emission factor databases and expand system boundaries to encompass the entire life cycle, thereby providing a more granular foundation for the soybean industry’s low-carbon transition.

### 4.4. Targeted Policy Implications and Future Pathways

The identification of distinct emission archetypes across China’s soybean-producing regions suggests that a “one-size-fits-all” mitigation strategy is suboptimal. Effective carbon management requires a transitioned focus from uniform administrative mandates to spatially tailored, precision interventions that address the specific input–output characteristics of each region. In fertilizer-driven and N_2_O-dominant regions, such as Shaanxi and Heilongjiang, where disproportionate emissions indicate that nitrogen surplus frequently exceeds soil retentive capacity, mitigation must prioritize precision nitrogen management to intercept reactive nitrogen before it converts into high-GWP gases.As evidenced by Wang et al. (2018) [38], regional nitrogen management optimized through soil testing can reduce emissions by up to 30% without compromising soybean protein content, representing a critical lever for high-latitude intensive systems [55,56].

In contrast, for Pesticide-Intensive Zones like the Huang-Huai-Hai Plain, the transition should focus on breaking the cycle of chemical dependency driven by high cropping indices and intense pest pressure. Moving beyond traditional application methods, policies should subsidize the deployment of biological control agents and AI-driven automated monitoring systems to facilitate a shift toward an Integrated Pest Management framework. This approach, as identified by Deutsch et al. (2018) [57], serves as a key driver in reducing the indirect carbon costs associated with chemical manufacturing and frequent field spraying operations. Meanwhile, in Diesel-Prominent Areas such as Inner Mongolia, the high relative contribution of fuel highlights a “mechanical energy trap” inherent in large-scale operations. Strategic pathways in these frontiers involve upgrading aging machinery fleets and incentivizing the transition to electric or low-emission equipment [58]. Research by Chu et al. (2021) underscores that the synergy between large-scale land consolidation and energy-efficient mechanization [48], particularly through the promotion of conservation tillage, is fundamental to achieving absolute emission reductions in high-mechanization regions.

Looking forward, while seed production currently contributes minimally to the total Carbon Footprint, it remains the ultimate technical frontier for structural de-carbonization [58]. We posit that the terminal solution to the soybean industry’s carbon challenge lies in bio-breeding. By leveraging advanced biotechnologies to enhance the endogenous nitrogen-fixing capacity of soybean varieties, and potentially developing genotypes with higher photosynthetic efficiency, the industry can decouple growth from chemical inputs at a molecular level. This long-term pathway transforms soybean from a carbon-intensive crop into a cornerstone of China’s “Green Grain Warehouse,” ensuring that food security and carbon neutrality are pursued as mutually reinforcing objectives rather than conflicting mandates.

## 5. Conclusions

This study provides a comprehensive decadal assessment of the carbon footprint of soybean production across China’s major producing provinces from 2014 to 2023. Our analysis reveals that while China’s soybean industry has successfully increased production capacity, the associated carbon intensity has entered a stage of “fluctuating optimization.” The observed downward trend in both area-based and yield-based Carbon Footprint indicates a significant improvement in low-carbon efficiency, the underlying drivers of which warrant further investigation.

The core scientific contribution of this research lies in uncovering the profound spatial heterogeneity and the “yield dilution effect” inherent in regional soybean systems. By identifying distinct emission structures—ranging from fertilizer-dominated systems in the Northeast to pesticide-intensive patterns in the Huang-Huai-Hai Plain—this study moves the academic discourse from generic national estimates toward a more nuanced, mechanism-based understanding of agricultural emissions. These findings demonstrate that a “one-size-fits-all” approach to carbon mitigation is insufficient; instead, regional strategies must be tailored to the specific input structures of each local production system.

Beyond the immediate results, our longitudinal framework offers a strategic roadmap for balancing soybean security with global “Double Carbon” commitments. Our findings are consistent with the notionthat the low-carbon transition in agriculture is not a linear path but a dynamic process susceptible to socioeconomic and climatic shocks. Ultimately, this study serves as a critical evidence base for policymakers to optimize agricultural resource allocation, ensuring that China’s pursuit of food self-sufficiency is harmonized with long-term environmental sustainability.

## Figures and Tables

**Table 1:** Emission Factor for Agricultural Inputs in Soybean Carbon Footprint Calculation.

Agricultural Input	Carbon Emission Factor (kg CO_2_eq·kg^−1^)	Source
Seeds	0.25	[29]
Nitrogen Fertilizers	1.53	[30]
Phosphorus Fertilizers	1.63	[30]
Potassium Fertilizers	0.66	[30]
Compound Fertilizers	1.77	[31,32]
Diesel	0.89	[33]
Pesticides	10.2	[30]

## Data Availability

The original contributions presented in the study are included in the article/Supplementary Material, further inquiries can be directed to the corresponding authors.

## Supplementary Materials

The following supporting information can be downloaded at: https://www.mdpi.com/article/10.3390/foods15111979/s1, Table S1. A comparison of the results between this study and previous studies (Carbon Footprint Estimates). Table S2. A comparison of the results between this study and previous studies (Component Contribution).
