# Supplementary material for: Spatiotemporal Analysis of the Carbon Footprint of Soybean Production in China Based on Life Cycle Assessment
Source: Foods. 2026 Jun 2;15(11):1979. doi: 10.3390/foods15111979 (PMC13257074; doi:10.3390/foods15111979)
Supplement: Supplementary file 1 [file foods-15-01979-s001.zip › foods-4239994-supplementary.pdf]

Supplementary Information (SI)

Spatiotemporal Analysis of the Carbon Footprint of Soybean Production in China Based on Life Cycle Assessment

This file includes:

Supplementary tables

Tables S1 to S2

Table S1. A comparison of the results between this study and previous studies (Carbon Footprint Estimates)

Sources	Time range	Region	Carbon Footprints per unit yield	Carbon Footprints per unit area
Ours	2014-2023	Heilongjiang	0.26	542
		Jilin	0.26	586
		Liaoning	0.25	579
[1]	2018	Nenjiang Farm, under the Heilongjiang Land Reclamation Area, China	0.51	1470
[2]	2011-2023	Heilongjiang	0.34	590
		Jilin	0.35	690
		Liaoning	0.29	700
[3]	2011-2020	Heilongjiang	0.337	/

Note: The system boundary and scope for the above research are identical. Boundary: Sowing, tillage, irrigation, fertilization, and harvesting. Scope: Soil-derived nitrous oxide (N_2O) emissions and carbon emissions associated with agricultural inputs.

Table S2. A comparison of the results between this study and previous studies (Component Contribution)

**Sources**	**Time range**	**Region**	**Components**	**Contribution to Carbon Footprint**
Ours	2014-2023	Henan	Chemical Fertilizer	24.59%
		The Three Northeastern Provinces (Heilongjiang, Jilin, Liaoning)	Chemical Fertilizer	52.34%
			Soil N_2O	15.78%
		Heilongjiang	Soil N_2O	21.49
[4]	2004-2022	Henan	Chemical Fertilizer	63.76%

Sources	Time range	Region	Components	Contribution to Carbon Footprint
[2]	2011-2023	The Three Northeastern Provinces (Heilongjiang, Jilin, Liaoning)	Chemical Fertilizer	45%
			Soil N_2O	16%
[3]	2011-2020	Heilongjiang	Soil N_2O	22.71%

References

1. Chu Tianshu, Lai Shixuan, Han Lujia, et al. Evaluation of Mechanized Soybean Production Models in China and Kazakhstan Using the Carbon Footprint Method [J]. Transactions of the Chinese Society of Agricultural Engineering, 2021, 37(03): 312–319.
2. Xiu Zhisheng, Li Yan, Wei Dan, et al., 2025, ‘A Study on the Dynamic Changes in the Carbon Footprint of Major Food Crops in the Three Northeastern Provinces and Their Influencing Factors’, Soil Bulletin, Vol. 56, pp. 27–36
3. Chen Hanyi, Chen Yiling, Hong Zhikun, et al. Spatiotemporal Distribution Characteristics of the Carbon Footprint and Eco-logical Optimization of Soybean Production in Heilongjiang Province [J]. Environmental Monitoring Management and Technology, 2024, 36(03): 21-26.
4. Li Jiangtao, Yu Huiyong, Guo Haiyue, et al. Carbon Footprint Analysis of Chemical Fertilizer Inputs in the Soybean Production System of Henan Province [J]. Soybean Science, 2025, 44(02): 85-92.
